# Spatiotemporal Metabolic Reprogramming of *Casuarina equisetifolia* in Response to an Interaction-Conditioned Filtrate of the Plant Growth-Promoting Bacterium LB46

**DOI:** 10.3390/plants15213469

**Published:** 2026-11-06

**Authors:** Wei Chu, Hezhi Lin, Jianjuan Li, Miao Jia, Shenghang Wang, Mingzhe Li, Sansan Liu, Zeyan Wu, Wenxiong Lin

**Affiliations:** 1 College of Life Sciences, Fujian Agriculture and Forestry University, Fuzhou 350002, China; 22305025005@fafu.edu.cn (W.C.);; 2 Key Laboratory of Crop Ecology and Molecular Physiology, Fujian Agriculture and Forestry University, Fuzhou 350002, China; 3 People’s Government of Fuqing City, Fuzhou 350002, China; 4 Fujian Academy of Forestry Survey and Planning, Fuzhou 350002, China

**Keywords:** plant growth-promoting rhizobacteria, interaction-conditioned filtrate, untargeted metabolomics, temporal and tissue-differentiated metabolic remodeling

## Abstract

Plant growth-promoting bacteria can influence host metabolism, but live-inoculation systems confound chemical signaling with colonization, nutrient exchange, and continuous metabolite release. Here, *Paenibacillus cellulositrophicus* LB46 was co-cultured with sterile *Casuarina equisetifolia* seedlings for 5 d. After 0.22 μm filtration, the interaction-conditioned filtrate was applied to new sterile seedlings; no culturable LB46 was detected in the filtrate under the plating conditions used. Untargeted LC–QTOF-MS profiled roots after 3 and 5 d and shoots after 5 d. The filtrate induced broad metabolic remodeling, with stronger responses in roots than in shoots. A total of 473, 437, and 125 metabolites met the differential-abundance criterion (BH-FDR < 0.05) in 3 d roots, 5 d roots, and 5 d shoots, respectively; 273 root metabolites showed a significant treatment × time interaction (BH-FDR < 0.05). Temporal classification revealed pronounced turnover among early-transient, late-induced, and sustained responses. At 5 d, only 11 metabolites met the differential-abundance criterion in both roots and shoots, indicating predominantly tissue-differentiated remodeling. These results define temporally and spatially differentiated metabolic responses to the interaction-conditioned environment. Because bulk physicochemical properties of the filtrates were not characterized, the observed responses cannot be attributed exclusively to specific chemical signals.

## 1. Introduction

Plant-associated microorganisms influence host performance through nutrient exchange, phytohormone modulation, and a diverse repertoire of extracellular metabolites. Increasing evidence indicates that these interactions are best understood as dynamic chemical exchanges rather than as a simple delivery of individual growth-promoting compounds [1]. Root-derived metabolites shape microbial activity, while microorganisms reciprocally modify host metabolism locally and systemically, creating a metabolically connected root–microbiome–shoot continuum [2,3,4]. Recent advances in plant microbiome research have therefore shifted attention from microbial taxonomic composition toward the biochemical processes that determine host responses and the consistency of microbial biostimulants [5,6].

Most studies of plant growth-promoting rhizobacteria (PGPR), however, rely on live inoculation and assess growth, nutrient acquisition, or stress-related traits at one or a few endpoints [7]. Such designs integrate bacterial colonization, continued metabolite production, nutrient competition, and host feedback, making it difficult to isolate the contribution of the chemical environment generated during plant–bacterium interaction. Cell-free bacterial supernatants can retain phytohormones, organic acids, peptides, siderophores, and other bioactive metabolites and can influence plant physiology in the absence of viable bacterial cells [8,9,10]. Yet a bacterial monoculture supernatant differs fundamentally from an interaction-conditioned filtrate produced during plant–bacterium co-culture: the latter may contain bacterial products, plant-derived compounds, and metabolites generated or transformed specifically during their interaction. The host metabolic consequences of such chemically conditioned environments remain poorly resolved, particularly with respect to their temporal organization and root-to-shoot distribution [11]. Importantly, an interaction-conditioned filtrate should not be equated with a purified signaling medium. In addition to potentially bioactive metabolites, it may contain residual medium constituents, transformation products, and co-culture-induced changes in nutrient availability, pH, ionic composition, or other physicochemical properties. Here, “interaction-conditioned filtrate” therefore refers to the composite filtered chemical environment generated during plant–bacterium co-culture, without assigning its effects to a specific chemical component or source.

*Casuarina equisetifolia* is a stress-tolerant actinorhizal tree widely used in coastal protection and ecological restoration. Its close association with root- and nodule-associated microorganisms makes it a useful woody system for studying beneficial plant–microbe interactions. We previously isolated strain LB46 from *C. equisetifolia* root nodules and identified it as *Paenibacillus cellulositrophicus*. LB46 displayed multiple plant growth-promoting traits, including nitrogenase activity, indole-3-acetic acid (IAA) production, ammonia production, cellulolytic activity, and siderophore production [12]. Its nitrogenase activity reached 188.33 ± 6.23 U mL^−1^ and IAA production 10.21 ± 1.11 mg L^−1^ in the original screening assay. Subsequent long-term inoculation experiments further showed that LB46-containing treatments altered *C. equisetifolia* growth and physiological responses within a temporally dynamic plant–soil–microbe system [13]. These studies established the biological relevance of LB46, but did not determine whether the chemical milieu generated during direct LB46–*C. equisetifolia* interaction is itself sufficient to reprogram host metabolism.

Here, we separated chemical conditioning from continued bacterial colonization by exposing new sterile *C. equisetifolia* seedlings to a cell-free filtrate generated during LB46–seedling co-culture. Untargeted LC–QTOF-MS metabolomics was then used to resolve root responses at 3 and 5 d and shoot tissue responses at 5 d. We addressed three questions. First, does the conditioned filtrate induce a persistent metabolic response, or does the root metabolome undergo temporal turnover between early and later stages? Second, is a comparable metabolic response detectable in shoot tissue, or does it remain tissue-differentiated? Third, which metabolic contexts recur across stage-, temporal, and tissue-resolved analyses? The 3 and 5 d sampling points were used to contrast earlier and later short-term responses and were not intended to resolve a complete kinetic sequence or establish temporal causality. Because shoots were sampled only at 5 d, earlier or later systemic responses could not be evaluated.

This study therefore characterizes the temporal and tissue-resolved response architecture of the conditioned filtrate treatment rather than identifying the causal chemical drivers of that response.

## 2. Results

### 2.1. Quality-Control Evidence Supports Reproducible Metabolomic Profiles Across Time and Tissues

Untargeted LC–QTOF-MS profiling yielded 4546 putatively annotated metabolite features, including 2314 detected in positive-ion mode and 2232 in negative-ion mode. Root and shoot tissue samples were prepared and acquired within the same LC–MS analytical batch, but were analyzed separately by tissue to resolve treatment-related variation against their distinct metabolic backgrounds. No missing, zero, or negative abundance values were detected. After log_2_ transformation, 49 zero-variance features were removed independently from each tissue dataset, leaving 4497 testable metabolites for downstream multivariate and differential analyses. Raw abundance distributions were strongly right-skewed, whereas log_2_ transformation substantially improved distributional symmetry without introducing missing values (Figure S1A–D). Sample-level total abundance showed no exceptional profiles, and no zero or missing values were detected before modeling (Figure S1E). Pairwise sample correlations and PCA scree profiles further supported high replicate reproducibility and a multivariate structure distributed across several principal components (Figure S2).

**Figure 1:**
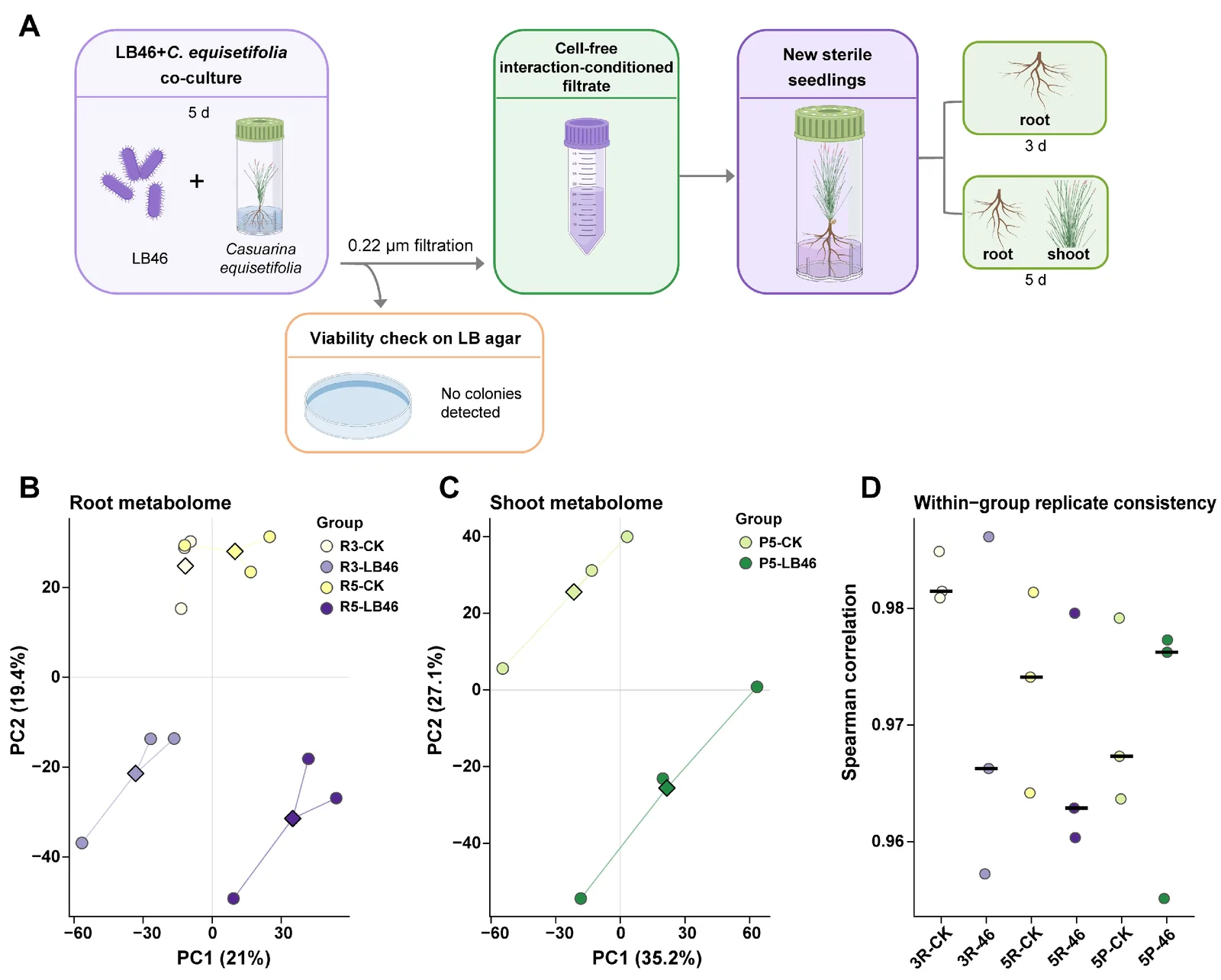
Experimental design, filtrate viability verification, and metabolomic quality assessment of LB46–*C. equisetifolia* interaction-conditioned filtrate responses. (**A**) Experimental workflow showing generation of the LB46–*C. equisetifolia* interaction-conditioned filtrate, verification of the absence of culturable bacteria, recipient-seedling treatment, and metabolomic sampling. LB46 was co-cultured with sterile *C. equisetifolia* seedlings for 5 d, after which the culture medium was centrifuged and passed through a 0.22 μm membrane filter. For each independently prepared filtrate, a 100 μL aliquot was plated on LB agar and incubated at 37 °C for 48 h; no colonies were detected. Independently prepared cell-free filtrates were subsequently applied to new sterile seedlings, and roots were sampled after 3 d (R3) and 5 d (R5), whereas shoots were sampled after 5 d (P5). The control arm is not depicted in panel (**A**). In panels (**B**–**D**), CK denotes recipients of the corresponding plant-conditioned control filtrate generated without LB46, whereas 46 denotes recipients of LB46-conditioned filtrate. (**B**) Principal component analysis (PCA) of the root metabolome using R3-CK, R3-LB46, R5-CK, and R5-LB46 samples. (**C**) PCA of the 5 d shoot tissue metabolome. Root and shoot tissue samples were acquired within the same LC–MS analytical batch but were analyzed separately by tissue to resolve treatment-associated variation within each organ. PCA was performed on log_2_-transformed, unit-variance-scaled abundance data after removal of zero-variance features. In panels (**B**) and (**C**), diamonds indicate group centroids, and circles represent individual biological replicates. (**D**) Within-group Spearman correlations among biological replicates. A total of 4546 putatively annotated metabolite features were retained as the primary annotation universe, and 4497 features per tissue were testable after removal of zero-variance variables. Black horizontal lines indicate the mean within-group Spearman correlation coefficients.

Biological replicates showed consistently high within-group reproducibility, with mean Spearman correlations ranging from 0.968 to 0.982 across the six treatment groups (Figure 1D). Principal component analysis further revealed group-structured variation across treatments and sampling stages. In roots, PC1 and PC2 explained 21.0% and 19.4% of the variance, respectively, whereas the corresponding components explained 35.2% and 27.1% of the shoot tissue variance (Figure 1B,C). No sample showed a reproducibility or abundance profile warranting exclusion.

Four pooled QC samples were included in the analytical sequence. Pairwise Spearman correlations among the QC samples ranged from 0.9876 to 0.9940. In addition, 17,128 of the 18,364 detected features (93.27%) and 4300 of the 4546 annotated features used in this study (94.59%) showed QC relative standard deviations (RSDs) ≤ 30%. QC samples also clustered tightly in QC-inclusive PCA analyses. Together, these quality-control metrics supported good analytical reproducibility during LC–QTOF-MS acquisition (Figure S4) and provided no indication of major analytical instability affecting the dataset.

### 2.2. Differential Metabolic Responses Remain Extensive in Roots but Contract Sharply in Shoot Tissue

**Figure 2:**
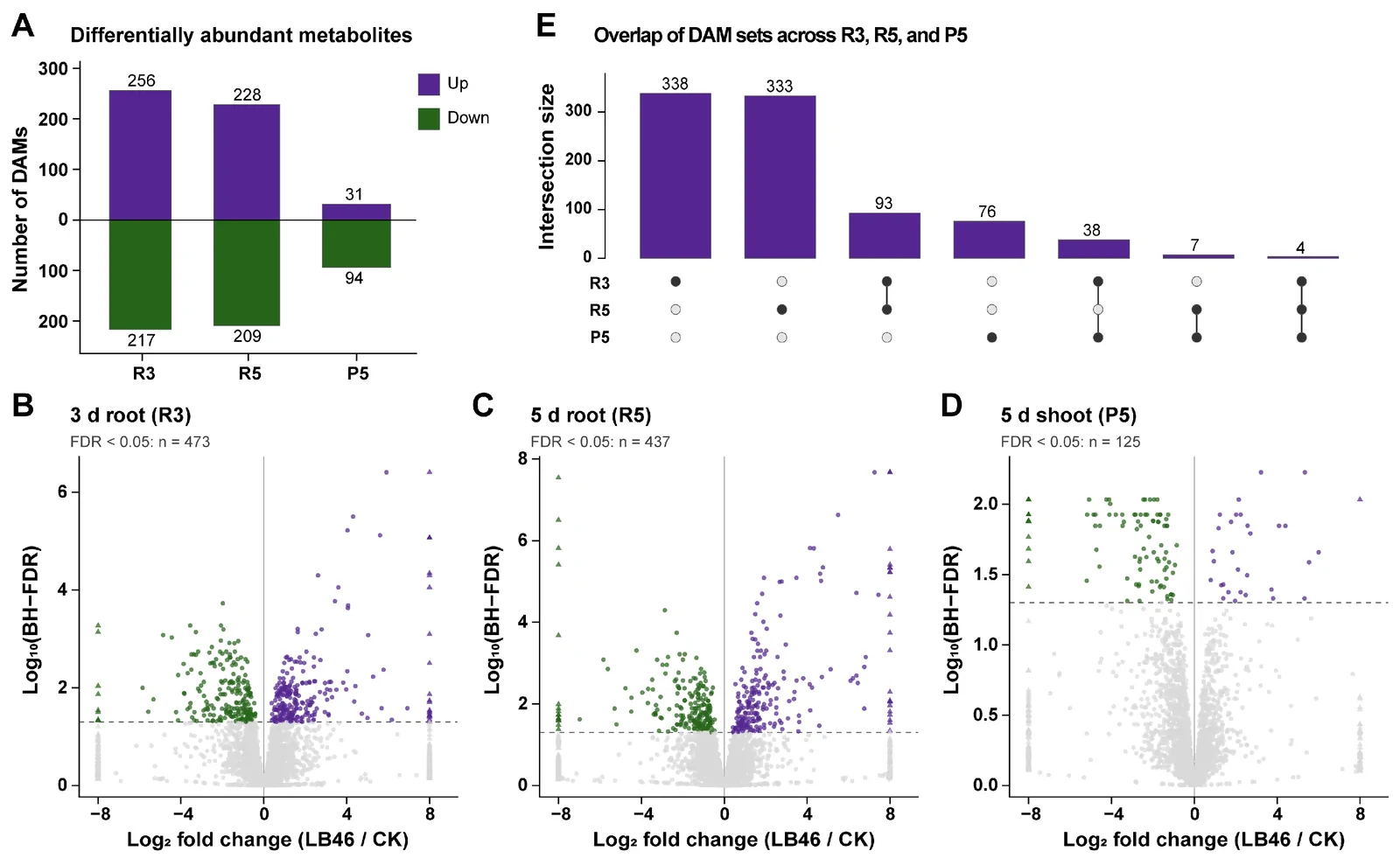
Differential metabolite landscapes reveal extensive root responses, temporal turnover, and a reduced shoot tissue response. (**A**) Numbers of increased and decreased differentially abundant metabolites (DAMs) at R3, R5, and P5. DAMs were defined by limma BH-FDR < 0.05 without an additional fold-change cutoff. (**B**–**D**) Volcano plots for R3, R5, and P5, respectively. Positive log_2_FC values indicate higher abundance under LB46-conditioned filtrate treatment relative to CK. Values beyond the displayed log_2_FC range were clipped for visualization only and are indicated by triangular symbols; all statistical analyses used the original values. The solid grey vertical line indicates log_2_(fold change) = 0, whereas the dashed grey horizontal line indicates the significance threshold (BH-FDR = 0.05). Grey dots represent metabolites that did not meet this significance threshold (BH-FDR ≥ 0.05). (**E**) Overlap of DAM sets across R3, R5, and P5. The UpSet plot shows exclusive and shared DAM sets among the three comparisons. A total of 889 unique DAMs were detected across R3, R5, and P5. The R3–R5-only intersection contains 93 metabolites, and four additional metabolites were significant in all three comparisons; hence, 97 metabolites were significant at both root time points (93 + 4). The union comprises 889 unique DAMs.

Using limma with BH-FDR < 0.05 and without imposing an arbitrary fold-change threshold, 473 differentially abundant metabolites (DAMs) were detected in roots at 3 d (R3), comprising 256 increased and 217 decreased features (Figure 2A,B). At 5 d, roots retained a comparably extensive response, with 437 DAMs (228 increased and 209 decreased; Figure 2A,C). By contrast, only 125 DAMs were detected in shoot tissue at 5 d (P5), of which 31 increased and 94 decreased, indicating a markedly smaller response dominated by decreases (75.2%; Figure 2A,D).

Across the three comparisons, 889 unique DAMs were detected. Despite the similar numbers of R3 and R5 DAMs, their compositions differed substantially: 338 were unique to R3 and 333 were unique to R5, whereas only 97 metabolites were significant at both root time points (Figure 2E). Among the remaining intersections, 38 DAMs were shared between R3 and P5 and seven between R5 and P5, and only four were common to all three comparisons. Thus, the similar overall magnitude of the root response at 3 and 5 d masked substantial turnover in the identity of responsive metabolites.

### 2.3. Root Metabolic Responses Undergo Pronounced Temporal Turnover from Early-Transient to Late-Induced Remodeling

Because similar numbers of root DAMs at R3 and R5 do not distinguish persistence from replacement, we next tested whether individual treatment effects changed across time using an explicit treatment × time contrast.

**Figure 3:**
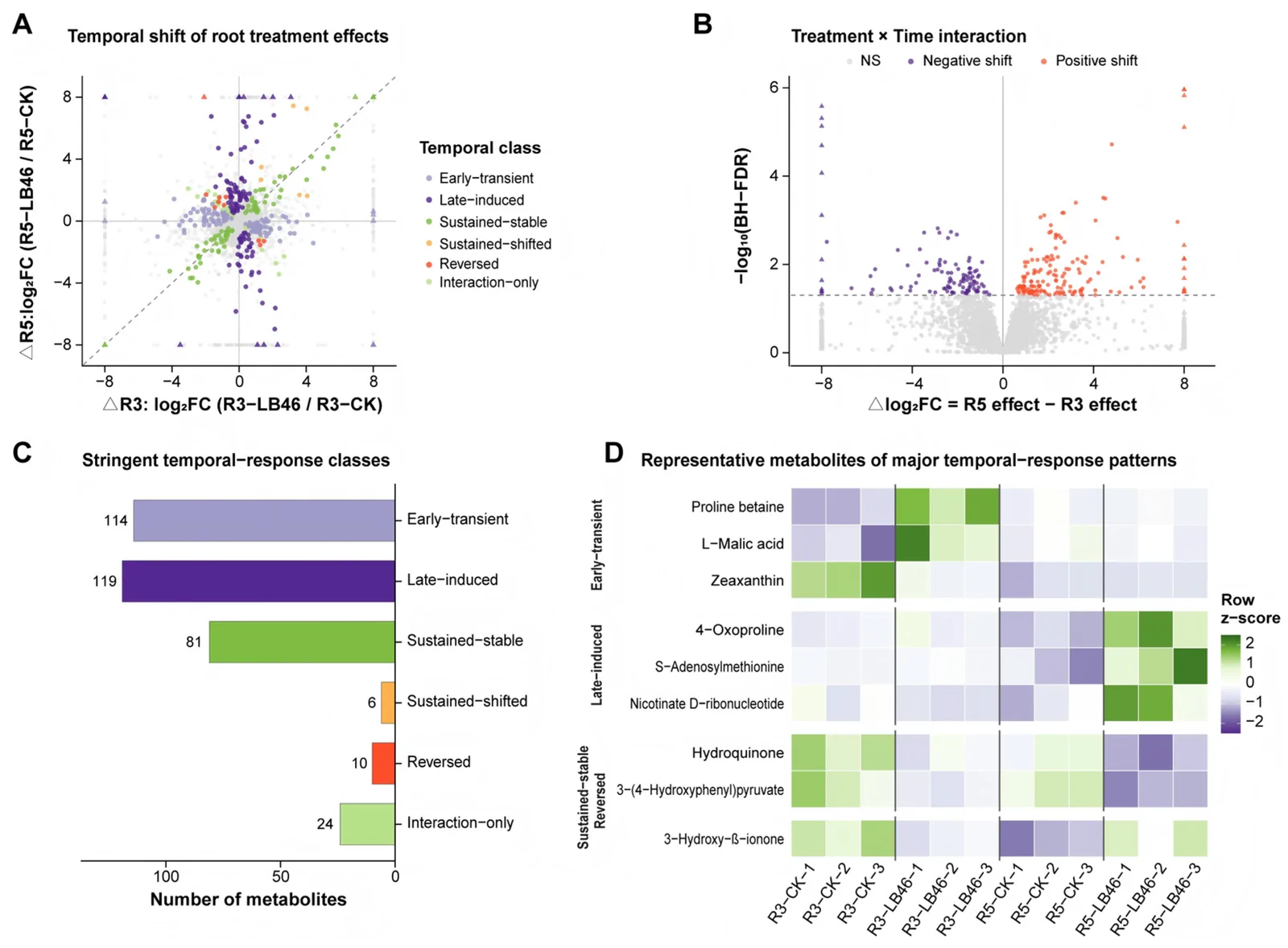
Temporal turnover reorganizes the root metabolic response from early-transient to late-induced patterns. (**A**) Comparison of treatment effects at R3 and R5. Each point represents one metabolite; colors denote temporal-response classes. The dashed diagonal denotes equal treatment effects at the two time points. Values beyond ±8 log_2_FC were clipped for visualization only. (**B**) Volcano plot of the treatment × time interaction contrast, calculated as the R5 treatment effect minus the R3 treatment effect. Positive values indicate a more positive LB46 effect at R5, whereas negative values indicate a more negative effect. A total of 273 metabolites showed BH-FDR < 0.05. In panels (**A**) and (**B**), triangles indicate metabolites with log_2_ fold-change values outside the displayed range (−8 to 8), plotted at the corresponding axis limits for visualization. (**C**) Numbers of metabolites assigned to six stringent temporal-response classes: early-transient, late-induced, sustained-stable, sustained-shifted, reversed, and interaction-only. (**D**) Row-z-score heatmap of nine representative metabolites illustrating the major temporal-response patterns across 12 root biological replicates. Metabolites were selected using differential abundance, interaction statistics, annotation plausibility, and pathway context.

To determine whether the root response changed quantitatively over time, treatment effects at R3 and R5 were directly compared using a treatment × time interaction contrast. A total of 273 metabolites showed a significant interaction (BH-FDR < 0.05), demonstrating that a substantial fraction of the root response changed between 3 and 5 d rather than simply persisting at a constant magnitude (Figure 3A,B).

Integration of the R3, R5, and interaction statistics resolved six temporal-response classes (Figure 3C). These included 114 early-transient, 119 late-induced, 81 sustained-stable, six sustained-shifted, 10 reversed, and 24 interaction-only metabolites. The 97 metabolites significant at both R3 and R5 were completely partitioned into 81 sustained-stable, six sustained-shifted, and 10 reversed responses, linking the set-overlap analysis in Figure 2 directly to the temporal interaction model.

Representative metabolites reproduced these distinct temporal patterns at the biological-replicate level (Figure 3D). Proline betaine increased strongly at R3 (log_2_FC = 3.43, FDR = 1.69 × 10^−4^) but showed essentially no treatment effect at R5 (log_2_FC = −0.05, FDR = 0.959; interaction FDR = 0.00191). L-malic acid similarly shifted from an R3 increase (log_2_FC = 1.05, FDR = 0.00958) to no detectable R5 response (log_2_FC = −0.10, FDR = 0.808; interaction FDR = 0.0389). In contrast, 4-oxoproline was predominantly late-induced, increasing at R5 by log_2_FC = 2.57 (FDR = 7.10 × 10^−4^), while S-adenosylmethionine and nicotinate D-ribonucleotide also became significant only at R5. Sustained decreases were observed for hydroquinone and 3-(4-hydroxyphenyl)pyruvate, whereas 3-hydroxy-β-ionone reversed from a decrease at R3 (log_2_FC = −1.18, FDR = 0.0205) to an increase at R5 (log_2_FC = 1.53, FDR = 0.00517; interaction FDR = 0.00313). These results establish a pronounced temporal turnover of the root metabolic response.

### 2.4. Five-Day Metabolic Remodeling Is Predominantly Tissue-Differentiated Rather than Broadly Shared

Having established substantial temporal turnover within roots, we next asked whether the later R5 metabolic state was mirrored in shoots.

To determine whether the 5 d root response was similarly expressed in shoot tissue, we compared within-tissue LB46-versus-control effect sizes rather than raw metabolite abundances. This design isolates treatment responsiveness from the large baseline metabolic differences expected between root and shoot tissue. Among metabolites with valid limma estimates in both tissues, 4452 features formed the common analytical universe. Of these, 429 were significant in roots and 125 in shoots. Treatment effect magnitudes showed negligible global concordance (Pearson *r* = 0.015, *p* = 0.323; Spearman *ρ* = 0.064).

**Figure 4:**
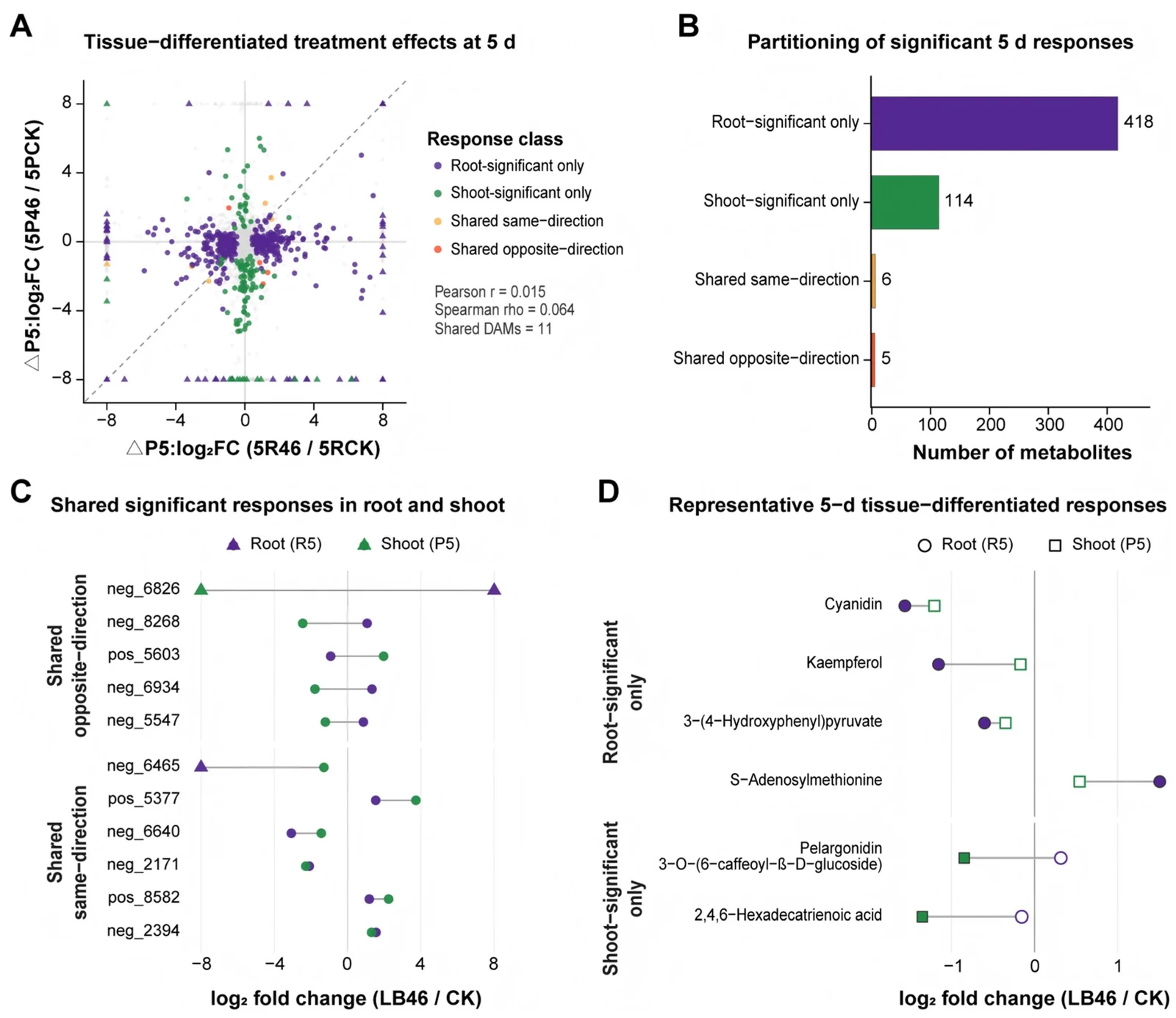
Five-day metabolic remodeling is dominated by tissue-differentiated rather than broadly shared responses. (**A**) Comparison of within-tissue treatment effects for the 4452 metabolites jointly testable in root and shoot tissue at 5 d. Effects are expressed as log_2_FC(LB46/CK). Although both tissues were acquired within the same LC–MS analytical batch, comparisons were based on within-tissue effect sizes rather than raw peak areas to evaluate concordance in treatment responsiveness. Triangles indicate metabolites with log_2_ fold-change values outside the displayed range (−8 to 8), plotted at the corresponding axis limits for visualization. (**B**) Partitioning of significant responses into root-significant-only, shoot-significant-only, shared-same-direction, and shared-opposite-direction classes. Eleven metabolites were significant in both tissues, compared with 12.05 expected under independence. (**C**) Root and shoot effect sizes for all 11 shared DAMs. The display range was fixed at ±8 log_2_FC; off-scale values were clipped to the boundary and marked by triangles, while original values were retained for all analyses. (**D**) Representative tissue-differentiated metabolites. Circles denote roots and squares denote shoot tissue; filled symbols indicate BH-FDR < 0.05 and open symbols indicate BH-FDR ≥ 0.05. “Significant only” refers to threshold status and does not imply the absence of an effect in the other tissue.

Restricting the analysis to the 543 metabolites significant in at least one tissue likewise yielded no significant association (Pearson *r* = −0.026, *p* = 0.552; Spearman *ρ* = 0.067, *p* = 0.122; Figure 4A). The significant responses were overwhelmingly tissue-differentiated: 418 metabolites were root-significant only, 114 were shoot-significant only, and only 11 were significant in both tissues (Figure 4B). The observed overlap was essentially identical to that expected under independence (12.05 metabolites; Fisher’s exact test, OR = 0.902, *p* = 0.878; Jaccard index = 0.020). Of the 11 shared DAMs, six changed in the same direction and five in opposite directions, providing no evidence for a consistent shared directional response (Figure 4C).

Selected metabolites further illustrated the tissue-differentiated pattern (Figure 4D). Cyanidin (R5 log_2_FC = −1.56, FDR = 0.0127), kaempferol (−1.15, FDR = 0.0390), 3-(4-hydroxyphenyl)pyruvate (−0.60, FDR = 0.00529), and S-adenosylmethionine (+1.51, FDR = 0.00460) reached significance only in roots. Conversely, pelargonidin 3-O-(6-caffeoyl-*β*-D-glucoside) and 2,4,6-hexadecatrienoic acid were significant only in shoot tissue (P5 log_2_FC = −0.85 and −1.35; FDR = 0.0196 and 0.0338, respectively). A sensitivity analysis based on limma moderated t-statistics produced only very weak cross-tissue concordance across all jointly testable metabolites (Pearson *r* = 0.068; Spearman *ρ* = 0.083) and across metabolites significant in at least one tissue (Pearson *r* = 0.090; Spearman *ρ* = 0.142). Although some correlations reached statistical significance because of the large number of metabolites tested, their small magnitudes were consistent with the predominantly tissue-differentiated response inferred from the log_2_FC analysis. Thus, the 5 d response was characterized primarily by tissue-differentiated rather than broadly shared metabolic remodeling.

### 2.5. KEGG Analysis Identifies Recurrent Pathway Tendencies Without Pathway-Level FDR Significance

The strong tissue differentiation raised the question of whether any recurrent biochemical contexts nevertheless emerged across independently defined temporal- and tissue-response sets.

**Figure 5:**
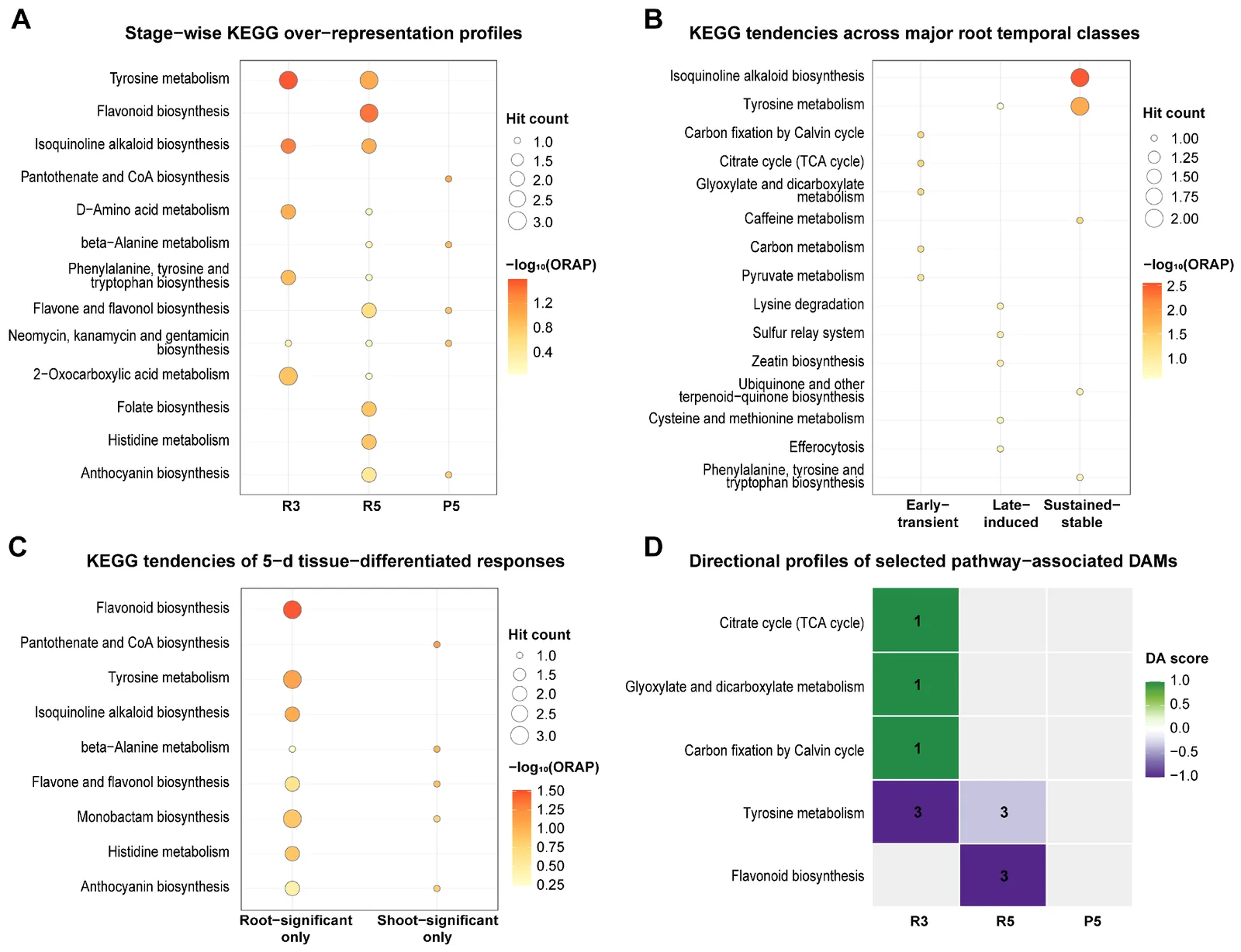
KEGG over-representation analysis provides pathway context for temporal and tissue-differentiated metabolic remodeling. (**A**) KEGG over-representation profiles of R3, R5, and P5 DAMs. (**B**) KEGG tendencies across the major root temporal-response classes. (**C**) KEGG tendencies associated with root-significant-only and shoot-significant-only responses at 5 d. In panels (**A**–**C**), bubble size represents the number of DAMs mapped to a pathway and bubble fill represents −log10(nominal ORA P). No pathway passed BH-FDR < 0.05, and the displayed patterns therefore represent exploratory pathway-level tendencies rather than statistically significant enrichment. (**D**) Directional abundance scores for five selected, biologically interpretable pathway contexts. DA score was calculated as (*N*_up_ − *N*_down_)/(*N*_up_ + *N*_down_); numbers within cells indicate pathway-associated DAM counts, and gray cells indicate no DAM assigned to the corresponding pathway at that stage. DA scores summarize the direction of pathway-associated DAMs and do not indicate biochemical pathway activation or inhibition. The R3 signals assigned to the TCA cycle, glyoxylate and dicarboxylate metabolism, and carbon fixation were all driven by the same L-malic acid hit and therefore do not represent three independent enrichment events.

KEGG over-representation analysis was recalculated using the limma-defined DAM sets. Pathway coverage was limited: only 26/473 R3, 40/437 R5, and 6/125 P5 DAMs carried KEGG pathway annotations. Similarly, 6/114 early-transient, 11/119 late-induced, 7/81 sustained-stable, 38/418 root-significant-only, and 5/114 shoot-significant-only metabolites were pathway-mapped. Because KEGG pathway annotations covered only a subset of the responsive metabolites, mapping coverage was explicitly quantified across stage-, temporal-, and tissue-response sets (Figure S3), providing the basis for the conservative interpretation of pathway-level signals. No KEGG pathway remained significant after BH correction in any comparison or response class (FDR < 0.05); pathway-level results were therefore treated as enrichment tendencies rather than formal significant enrichments (Figure 5A–C).

Several tendencies nevertheless recurred across independent response partitions. Flavonoid biosynthesis was represented among both R5 DAMs (three hits; enrichment ratio = 3.70, nominal *p* = 0.0343) and root-significant-only DAMs (three hits; enrichment ratio = 3.86, nominal *p* = 0.0306), consistent with the root decreases in cyanidin and kaempferol. Tyrosine metabolism was represented at R3 (three hits, nominal *p* = 0.0251) and among sustained-stable metabolites (two hits, nominal *p* = 0.0126), including hydroquinone and 3-(4-hydroxyphenyl)pyruvate. In the early-transient class, the TCA cycle, glyoxylate and dicarboxylate metabolism, and carbon fixation each showed nominal enrichment signals; however, all three were driven by the same L-malic acid hit and therefore represented a shared malate-centered annotation pattern rather than independent pathway events. No comparable nominally enriched pathway pattern was detected for P5 or the shoot-significant-only set. Directional abundance scores further summarized the predominant direction of DAMs within these selected pathway contexts without implying pathway activation or inhibition (Figure 5D).

### 2.6. Integrated Core-Metabolite Evidence Consolidates Temporal Turnover and Tissue Differentiation

**Figure 6:**
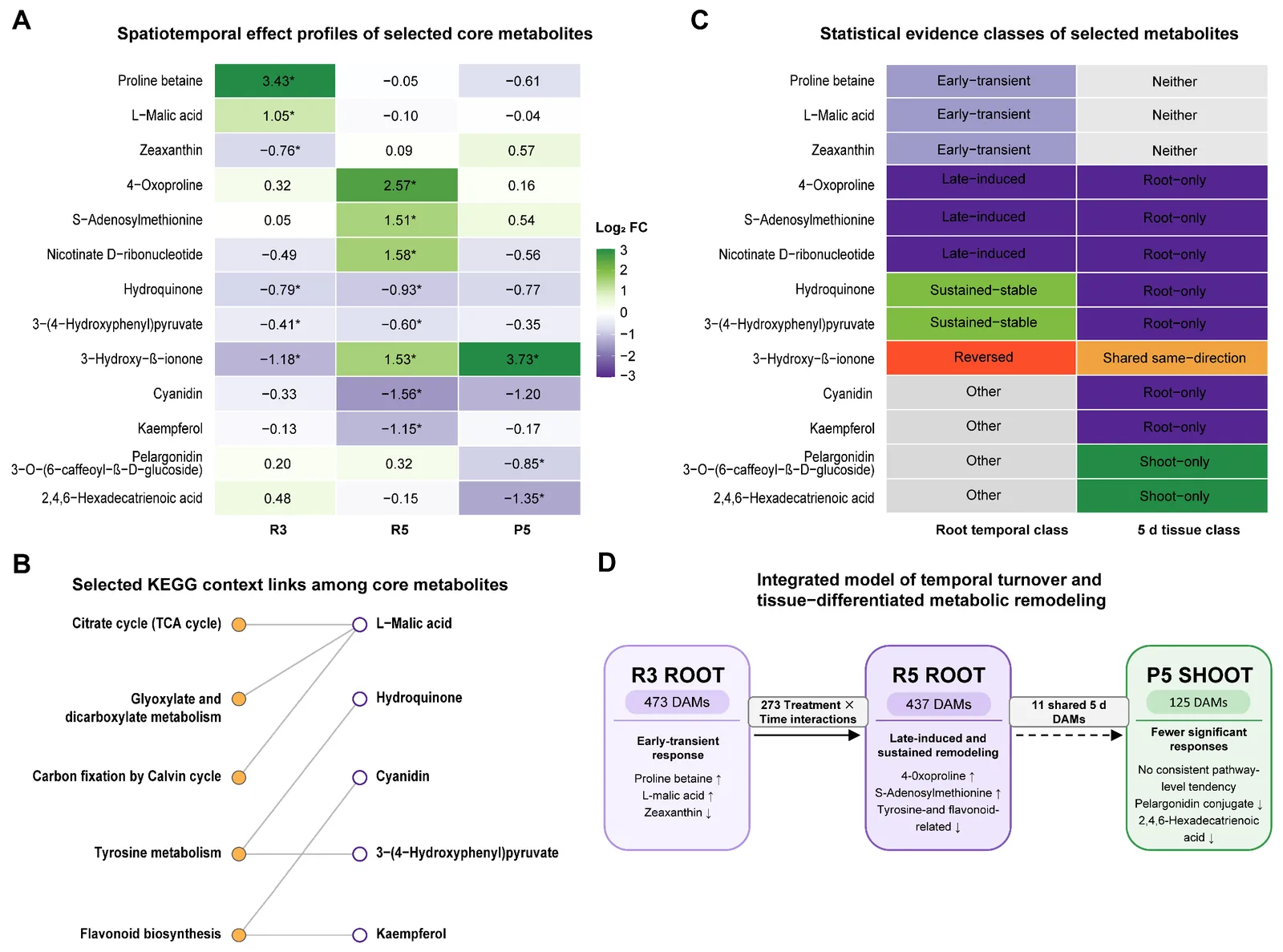
Temporal turnover and tissue differentiation characterize responses to LB46–*C. equisetifolia* interaction-conditioned filtrate. (**A**) Treatment effect profiles of 13 representative metabolites across R3, R5, and P5. Values indicate log_2_FC(LB46/CK); asterisks denote BH-FDR < 0.05. The heatmap color scale was clipped at |log_2_FC| = 3 for visualization only. (**B**) Selected KEGG annotation links between representative metabolites and the five retained pathway contexts. These links indicate annotation context only; no KEGG pathway passed BH-FDR < 0.05. (**C**) Statistical evidence matrix showing the root temporal-response class and 5 d tissue-response class assigned to each representative metabolite. Colors distinguish the different statistical evidence classes for root temporal responses and 5 d tissue-specific responses, as labeled within the cells. (**D**) Integrated evidence model summarizing the transition from early root responses at R3 to late/sustained root remodeling at R5 and the comparatively limited shoot tissue response at P5. Across stage-specific analyses, 473, 437, and 125 DAMs were detected at R3, R5, and P5, respectively; 273 metabolites showed significant treatment × time interactions. Within the 4452 metabolites jointly testable at 5 d, 418 were root-significant only, 114 were shoot-significant only, and 11 were significant in both tissues. The solid horizontal arrow between panels indicates the temporal comparison of root metabolic responses between 3 and 5 d, whereas the dashed horizontal arrow indicates the comparison between root and shoot responses at 5 d. Upward (↑) and downward (↓) arrows next to metabolite names indicate increased and decreased relative abundances, respectively, in LB46-treated seedlings compared with the corresponding controls. The horizontal arrows denote analytical comparisons rather than demonstrated metabolite transport or causal relationships. The model summarizes statistical evidence and does not imply metabolite transport, pathway activation, or direct causality. Metabolite identities derived from untargeted LC–QTOF-MS annotation remain putative unless independently validated.

Because pathway-level significance was limited by sparse KEGG coverage, we next integrated metabolite-level temporal, tissue and annotation evidence rather than inferring pathway activation. Integration of the temporal, tissue, and pathway analyses yielded 13 nonredundant representative metabolites that captured the principal response patterns across R3, R5, and P5 (Figure 6A). Their effect profiles recapitulated the major trends identified independently in Figure 3 and Figure 4: early-transient changes were concentrated at R3, late-induced responses emerged predominantly at R5, several metabolites showed sustained or reversed root responses, and two representative metabolites were significant only in shoot tissue.

Among these 13 metabolites, five were connected to the five retained KEGG pathway contexts through seven annotation links (Figure 6B). L-malic acid mapped to the TCA cycle, glyoxylate and dicarboxylate metabolism, and carbon fixation; hydroquinone and 3-(4-hydroxyphenyl)pyruvate mapped to tyrosine metabolism; and cyanidin and kaempferol mapped to flavonoid biosynthesis. The statistical evidence matrix further separated temporal-response classes from 5 d tissue-response classes, showing that early-transient metabolites generally lacked a significant 5 d tissue effect, whereas late-induced and sustained root responses were predominantly root-significant only (Figure 6C).

The integrated evidence model summarized the overall response architecture (Figure 6D). Across the complete stage-specific analyses, 473, 437, and 125 DAMs were detected at R3, R5, and P5, respectively, while 273 metabolites showed significant treatment × time interactions in roots. Within the 4452 metabolites jointly testable at 5 d, 418 were root-significant only, 114 were shoot-significant only, and only 11 were shared. Collectively, these results define a response characterized by pronounced temporal turnover in roots followed by strongly tissue-differentiated metabolic remodeling at 5 d.

## 3. Discussion

Together, the data reveal two defining properties of the conditioned filtrate response: pronounced temporal turnover within roots and limited concordance between root and shoot tissue remodeling.

The root response to the LB46–*C. equisetifolia* interaction-conditioned filtrate was strongly time-dependent. Comparable numbers of DAMs at 3 and 5 d therefore did not represent persistence of a fixed metabolic state; instead, extensive metabolite replacement and significant treatment × time effects revealed a transition from early-transient to late-induced and sustained responses. This distinction is important because biostimulant responses are often interpreted from single time points, although recent metabolomics studies increasingly show that microbial treatments generate sequential rather than static metabolic states [14]. In particular, *Paenibacillus alvei* T22 altered central carbon, aromatic amino-acid and phenolic metabolism in wheat in a treatment- and time-dependent manner [14].

This system further differs from conventional live-inoculation experiments because the recipient seedlings received a cell-free interaction-conditioned filtrate. Thus, recipient exposure was initiated with filtrates in which no culturable LB46 was detected under the plating conditions used. Recent assessments of bacterial cell-free supernatants show that metabolite-rich filtrates can modulate plant physiology independently of viable cells, although their activity depends strongly on strain and culture context [8,11]. However, the temporal pattern cannot be assigned specifically to a sequence of signal perception followed by host-derived metabolic reorganization. Several non-exclusive processes could contribute to the differences between 3 and 5 d, including host metabolic adjustment, depletion or uptake of filtrate constituents, metabolite degradation or transformation, nutrient depletion, and changes in pH, ionic composition, or osmotic conditions. Because these physicochemical variables were not measured, their contributions cannot be separated from metabolite-mediated effects in the present experiment.

The transient behavior of putatively annotated L-malic acid and the later emergence of changes associated with aromatic and specialized metabolism are consistent with temporal reorganization, but they do not establish a causal chain [15]. With only two root sampling times, the onset, peak, and duration of individual responses cannot be resolved. A denser time series would be required to establish temporal ordering. The key prediction is temporal: if early central-metabolic adjustment contributes to later reprogramming, perturbing malate metabolism or transport should alter at least part of the subsequent response.

At 5 d, the metabolic response was dominated by tissue differentiation rather than broad root–shoot concordance. This pattern is biologically plausible because the root was the tissue directly exposed to the conditioned filtrate. In tea, arbuscular mycorrhizal inoculation produced stronger metabolic reprogramming in roots than in shoot tissue, resembling the root-dominant pattern observed here [16]. By contrast, Jeon et al. [17] showed that live growth-promoting rhizobacteria extensively remodeled shoot tissue chemistry across several plant–bacterium combinations. These comparisons indicate that the spatial distribution of metabolic responses can vary among plant–microbe interaction systems, but the present data do not identify the mechanism responsible for the root–shoot difference. Because shoots were analyzed only at 5 d, an earlier or later systemic response cannot be excluded.

This contrast points to interaction mode as a likely determinant of spatial response. These comparisons indicate that the spatial distribution of metabolic responses can vary among plant–microbe interaction systems, but the present data do not identify the mechanism responsible for the root–shoot difference. Live rhizobacteria continuously participate in colonization, nutrient exchange and bidirectional chemical signaling, whereas the conditioned filtrate used here represents a non-renewing chemical input at the time of application. Beneficial plant–microbe interactions are now viewed as reciprocal chemical dialogues in which both partners continually modify the signaling environment [5,18].

Moreover, “root-significant only” should not be interpreted as absolute root specificity. Several metabolites changed in the same direction in both tissues but crossed the statistical threshold only in roots. The observed pattern is therefore better described as a quantitatively compartmentalized, root-biased response rather than evidence for a proven root-gating mechanism. The anatomical organization of *C. equisetifolia* roots provides a plausible biological context for such compartmentalization. Previous anatomical analysis has identified distinct epidermal, exodermal, endodermal, and stelar tissues in *C. equisetifolia* roots [19]. More generally, exodermal and endodermal barriers restrict radial movement of water and solutes and regulate access to the vascular tissues [20]. These structural features could therefore contribute to differential exposure, retention, or transport of filtrate-associated compounds between roots and shoots. However, root anatomy and solute transport were not measured in the present study, so this possibility remains hypothetical.

Direct testing of a root-gating mechanism would require additional spatially and temporally resolved experiments, including matched root and shoot sampling, xylem-sap analysis, and appropriate source- and medium-resolved controls.

At the pathway level, no KEGG pathway passed FDR correction, so the strongest pathway-level information lies in recurrent metabolic contexts rather than claims of pathway activation. This distinction is particularly important in a non-model woody species, where specialized metabolites vary substantially among taxa and tissues and many functional assignments remain incomplete [21,22].

Within this evidence boundary, three contexts recur coherently. First, the early malate-associated pattern links the initial response to central carbon metabolism [15]. Second, sustained changes in hydroquinone and 3-(4-hydroxyphenyl)pyruvate place tyrosine-associated metabolism within the persistent root response. Tyrosine and 4-hydroxyphenylpyruvate connect primary metabolism with diverse specialized products, and this branch is strongly lineage-dependent [23]. Its relevance to *C. equisetifolia* is strengthened by the abundant accumulation of the KEGG annotation context tyramine in mature *C. equisetifolia* nodules [24].

Third, the later decline in cyanidin and kaempferol places flavonoid metabolism within the root-biased 5 d response. This direction is consistent with the study of Jeon et al. [17], in which suppression of flavonoid biosynthesis was associated with rhizobacteria-mediated growth promotion. However, it contrasts with both AMF-treated tea, where flavonoids increased [6], and actinorhizal symbioses in which flavonoid accumulation and flavonoid-biosynthetic gene activation have been reported [25,26]. These apparently different outcomes suggest that flavonoid regulation is interaction-specific rather than universally positive or negative [27].

Accordingly, malate, tyrosine-associated metabolites and flavonoids should not be arranged as a linear biochemical pathway. A more defensible model is layered metabolic allocation: an early central-metabolic adjustment is followed by sustained aromatic-metabolic rebalancing and later root-biased remodeling of specialized-metabolite pools. Notably, the recent *P. alvei* T22 study also connected central carbon, aromatic amino-acid and phenolic metabolism, although the directions of individual metabolites differed [14]. This comparison suggests that the modules may recur across beneficial-microbe systems, whereas their direction and magnitude remain host- and interaction-dependent.

Together, the data define a root-centered response framework characterized by temporal turnover in roots and limited root–shoot concordance at 5 d. This framework describes the organization of the observed metabolic responses rather than a biochemical relay or signaling cascade.

Several limitations define the resolution of the present evidence. First, metabolite identities derived from untargeted LC–QTOF-MS were not confirmed with authentic reference standards [22]. Prominent assignments such as L-malic acid and kaempferol therefore remain putative. Second, pH, electrical conductivity, nutrient and ion concentrations, and other bulk physicochemical properties were not measured in the conditioned filtrates. Because recipient seedlings were exposed directly to non-renewed filtrates without fresh WPM, physicochemical changes cannot be separated experimentally from metabolite-mediated effects. Third, bacterium-only and untreated-medium controls were not included, preventing source attribution of individual chemical components.

The plating assay detected no culturable LB46 under the verification conditions used, but no molecular assay was performed to assess the presence of possible uncultured bacterial cells. In addition, the metabolomic experiment used three independent biological replicates per group. Although within-group reproducibility was high, this sample size limits statistical power, particularly for the small set of metabolites shared between roots and shoots. Future studies combining source-resolved and physicochemically characterized media, authentic-standard validation, denser temporal sampling, and paired root–shoot or root–xylem–shoot analyses will be required to identify causal chemical drivers, while comparisons between the single-strain LB46 system and defined phytobiome-derived consortia containing at least one additional bacterial isolate could further determine whether microbial community composition alters the temporal and tissue-resolved metabolic response patterns.

## 4. Materials and Methods

### 4.1. Bacterial Strain and Previous Characterization

*Paenibacillus cellulositrophicus* LB46 was originally isolated from surface-sterilized root nodules of *C. equisetifolia*. Its isolation, taxonomic identification, and plant growth-promoting characteristics have been described previously. LB46 exhibits multiple plant growth-promoting traits, including nitrogen fixation, phosphate solubilization, indole-3-acetic acid (IAA) production, ammonia production, cellulolytic and proteolytic activities, and siderophore production. In the initial screening assay, its nitrogenase activity and IAA production reached 188.33 ± 6.23 U mL^−1^ and 10.21 ± 1.11 mg L^−1^, respectively.

The present study did not repeat these strain-characterization assays. Instead, it focused on the metabolic response of *C. equisetifolia* to the chemical environment generated during direct LB46–seedling interaction.

### 4.2. Preparation of the LB46–C. equisetifolia Interaction-Conditioned Filtrate

Three-month-old sterile *C. equisetifolia* seedlings were used to establish the co-culture system. Each culture vessel contained one sterile seedling in 50 mL of liquid Woody Plant Medium (Qingdao Hope Bio-Technology, Qingdao, China; calcium nitrate was supplemented separately). LB46 was precultured in LB broth (Qingdao Hope Bio-Technology, Qingdao, China) for 1 d, after which the culture was adjusted to an OD_600_ of approximately 0.5. A 100 μL aliquot of the adjusted LB46 culture was added to each treatment vessel. Control vessels received an equal volume (100 μL) of sterile LB broth from the corresponding medium batch.

Cultures were maintained statically for 5 d in a controlled-climate growth chamber (Shanghai Yiheng Scientific Instrument Co., Ltd., Shanghai, China) under a 16 h light/8 h dark photoperiod. During the light period, the temperature was maintained at 30 °C, light intensity at 350 μmol m^−2^ s^−1^, and relative humidity at 60%; during the dark period, the temperature was maintained at 28 °C and relative humidity at 80%. All other culture conditions were identical between the treatment and control groups.

After 5 d, the culture media were collected and centrifuged at 12,000 rpm for 20 min at 4 °C. The resulting supernatants were passed through 0.22 μm membrane filters to remove bacterial cells and suspended particles. To verify the absence of culturable bacteria after filtration, 100 μL of filtrate from each individual culture vessel was spread onto LB agar and incubated at 37 °C for 48 h. No colony formation was observed for any filtrate used in subsequent experiments. No molecular assay was performed to assess the possible presence of nonculturable bacterial cells; therefore, the plating assay was used only to verify the absence of detectable culturable LB46 under the stated conditions. The cell-free filtrate obtained from the LB46–seedling co-culture is hereafter referred to as the LB46–*C. equisetifolia* interaction-conditioned filtrate. Control filtrates were generated from sterile seedlings cultured in WPM without LB46 and were subjected to the same incubation, centrifugation, filtration, and agar-plating verification procedures.

### 4.3. Recipient-Seedling Treatment and Tissue Sampling

A separate set of three-month-old sterile *C. equisetifolia* seedlings that had not previously been exposed to LB46 was used as recipient plants. Each recipient seedling was transferred to an independent sterile culture vessel containing 40 mL of the 0.22 μm filtered interaction-conditioned filtrate, with no fresh WPM added during the recipient treatment. No pH adjustment, nutrient replenishment, or physicochemical normalization was performed before recipient-seedling exposure. The pH, electrical conductivity, nutrient concentrations, and ion composition of the treatment and control filtrates were not measured. Accordingly, the comparison represents exposure to the complete conditioned media rather than to media standardized for physicochemical composition. Treatment seedlings received the LB46–*C. equisetifolia* interaction-conditioned filtrate, whereas control seedlings received the corresponding plant-conditioned control filtrate.

Each biological replicate represented an independent donor-filtrate-recipient experimental unit. The three biological replicates within each treatment group received filtrates generated independently from three separate donor co-culture vessels (or the corresponding independent control vessels) and were applied to three separate recipient seedlings. Thus, each experimental group contained three independent biological replicates *(n* = 3), rather than technical subdivisions of a common filtrate batch. Recipient seedlings were maintained under the same controlled-climate conditions described in Section 4.2 throughout the treatment period.

Roots were harvested after 3 d (R3) and 5 d (R5) of exposure, whereas shoot tissues were collected after 5 d (P5). The six experimental groups were therefore R3-CK, R3-LB46, R5-CK, R5-LB46, P5-CK, and P5-LB46. Harvested tissues were immediately frozen in liquid nitrogen and stored at −80 °C until metabolomic analysis.

### 4.4. Metabolite Extraction, LC–QTOF-MS Analysis, and Metabolite Annotation

#### 4.4.1. Metabolite Extraction

Frozen root and shoot tissues were processed using the same extraction procedure for untargeted metabolomic profiling. Briefly, 50 mg of frozen tissue was weighed and mixed with 1000 μL extraction solvent consisting of methanol (Merck KGaA, Darmstadt, Germany), acetonitrile (Merck KGaA, Darmstadt, Germany), and water at a ratio of 2:2:1 (*v*/*v*/*v*). The extraction solvent contained 20 mg L^−1^ 2-chloro-L-phenylalanine (Aladdin, Shanghai, China) as an internal standard [28].

Samples were vortexed for 30 s, mixed with steel beads, and homogenized at 45 Hz for 10 min, followed by 10 min of sonication in an ice-water bath. Extracts were then incubated at −20 °C for 1 h and centrifuged at 12,000 rpm for 15 min at 4 °C.

A 500 μL aliquot of the supernatant was transferred to a clean microcentrifuge tube and dried under vacuum. The dried extract was reconstituted in 160 μL acetonitrile/water (1:1, *v*/*v*), vortexed for 30 s, and sonicated for 10 min in an ice-water bath. After centrifugation at 12,000 rpm for 15 min at 4 °C, 120 μL of the supernatant was transferred to a 2 mL autosampler vial.

For quality-control (QC) analysis, 10 μL from each processed sample was pooled to generate a composite QC sample.

#### 4.4.2. LC–QTOF-MS Analysis

Untargeted metabolomic profiling was performed using a Waters ACQUITY UPLC I-Class PLUS system coupled to a Waters Xevo G2-XS quadrupole time-of-flight mass spectrometer (QTOF-MS; Waters Corporation, Milford, MA, USA). Chromatographic separation was achieved using an ACQUITY UPLC HSS T3 column (1.8 μm, 2.1 × 100 mm; Waters).

For both positive- and negative-ion modes, mobile phase A consisted of 0.1% formic acid (Tokyo Chemical Industry Co., Ltd., Tokyo, Japan) in water, and mobile phase B consisted of 0.1% formic acid in acetonitrile. The flow rate was maintained at 0.4 mL min^−1^, and the injection volume was 2 μL.

The chromatographic gradient used for LC–QTOF-MS analysis is shown in Table 1.

Mass-spectrometric data were acquired using MassLynx V4.2 (Waters) in MSE acquisition mode, enabling simultaneous collection of low- and high-collision-energy data within each acquisition cycle. No additional collision energy was applied in the low-energy channel, whereas the high-energy channel used a collision-energy ramp of 10–40 V. The scan time was 0.2 s per spectrum.

Electrospray ionization (ESI) parameters were as follows: capillary voltage, 2.5 kV in positive-ion mode and −2.0 kV in negative-ion mode; cone voltage, 30 V; source temperature, 100 °C; desolvation temperature, 500 °C; cone gas flow, 50 L h^−1^; and desolvation gas flow, 800 L h^−1^. Data were acquired over an *m*/*z* range of 50–1200.

Composite QC samples were analyzed periodically throughout the analytical sequence to monitor instrumental stability and analytical reproducibility. Four pooled QC injections were included. Pairwise Spearman correlations among the QC samples ranged from 0.9876 to 0.9940. Using RSD = sample standard deviation (n − 1)/mean × 100% across the four QC injections, 17,128 of the 18,364 detected features (93.27%) and 4300 of the 4546 annotated features used in this study (94.59%) showed QC RSD ≤ 30%. QC-inclusive PCA further showed tight clustering of the QC injections. These metrics were used to assess overall analytical reproducibility; no formal run-order drift model was applied.

#### 4.4.3. Data Preprocessing and Metabolite Annotation

Raw LC–QTOF-MS data acquired using MassLynx V4.2 were processed in Progenesis QI for peak detection, retention-time alignment, and peak-area integration. Metabolite annotation was performed by matching detected features against the online METLIN database, other public databases, and an in-house spectral library, together with theoretical fragment information.

Peak areas were used as measures of relative metabolite abundance. Accordingly, the dataset represents relative/semiquantitative rather than absolute quantification. Throughout the manuscript, metabolite identities are therefore described as putatively annotated metabolites unless independently confirmed using authentic standards [22]. No metabolite assignment, including L-malic acid and kaempferol, was independently confirmed by authentic-reference-standard retention time and MS/MS matching in the present study. The reported identities are therefore treated as putative annotations, broadly corresponding to Schymanski confidence Levels 2–3 depending on the available spectral evidence, rather than definitive Level 1 identifications.

### 4.5. Data Preprocessing and Quality Control

Root and shoot samples were prepared and analyzed within the same LC–MS analytical batch. For downstream statistical analyses, however, the abundance matrices were partitioned by tissue and evaluated separately. This strategy was used because root and shoot tissue have intrinsically different metabolic compositions. In addition, LC–QTOF-MS peak areas provide relative/semiquantitative measurements; therefore, raw peak areas were not used for direct quantitative comparisons between root and shoot tissue.

The primary annotation universe comprised 4546 putatively annotated metabolite features. No missing, zero, or negative abundance values were detected.

Because the raw peak-area distributions were strongly right-skewed, abundance values were transformed as “log_2_(*x* + *c*)” where *c* was defined as one-half of the minimum positive abundance in the dataset: *c* = 1.29265 × 10^−9^.

Given the absence of zero values, this pseudocount had a negligible influence on the observed abundance values.

For zero-variance filtering, each of the 4546 annotated features was evaluated separately across all 12 root samples (R3-CK, R3-LB46, R5-CK, and R5-LB46; *n* = 3 per group) and all six shoot biological samples (P5-CK and P5-LB46; *n* = 3 per group), following log_2_ transformation. The four pooled QC injections were used for analytical reproducibility assessment and were not included in biological zero-variance filtering. A feature was excluded from a tissue matrix if its transformed abundance was constant across every biological sample in that tissue. Forty-nine zero-variance features were removed independently from the root and shoot matrices, leaving 4497 testable features per tissue. Four features had zero variance in both tissue datasets, whereas 45 additional features were excluded only from roots and 45 only from shoots. Thus, 94 unique features were excluded from at least one tissue dataset, leaving 4452 features (4546 − 94) jointly testable in both tissues.

The present reanalysis used the abundance matrices exported by the metabolomics service provider. No additional total-ion-current, total-intensity, total-peak-area, internal-standard, or quantile normalization was applied during the present R-based reanalysis. Unit-variance scaling was not applied before differential-abundance modeling.

Biological-replicate consistency was assessed using Spearman rank correlations calculated from log_2_-transformed abundance values. PCA was performed independently for root and shoot tissue after centering and unit-variance scaling of the log_2_-transformed matrices.

### 4.6. Differential-Abundance Analysis and Treatment × Time Modeling

Differential-abundance analyses were performed in R version 4.3.0 using the limma empirical-Bayes framework [29].

Root samples were analyzed using a no-intercept group-mean model comprising R3-CK, R3-LB46, R5-CK, and R5-LB46. The treatment contrasts were defined as

*R*3 = *R*3_LB46_ − *R*3_CK_, *R*5 = *R*5_LB46_ − *R*5_CK_, (1)

and

Treatment × Time = (*R*5_LB46_ − *R*5_CK_) − (*R*3_LB46_ − *R*3_CK_) (2)

Shoot tissue samples were modeled independently, with the 5 d treatment effect defined as

*P*5 = *P*5_LB46_ − *P*5_CK_, (3)

Positive log_2_ fold-change (log_2_FC) values indicate higher relative abundance in seedlings treated with the LB46–*C. equisetifolia* interaction-conditioned filtrate than in the corresponding controls, whereas negative values indicate lower abundance.

For the interaction contrast, a positive coefficient indicates that the treatment effect became more positive, or less negative, from 3 to 5 d, whereas a negative coefficient indicates a shift in the opposite direction.

Empirical-Bayes moderation was performed using eBayes (trend = TRUE, robust = FALSE). Multiple-testing correction was conducted using the Benjamini–Hochberg procedure. Metabolites with BH-FDR < 0.05 were defined as differentially abundant metabolites (DAMs). No additional fold-change threshold was imposed for DAM definition [30].

### 4.7. Temporal-Response Classification and Comparison of 5 d Root and Shoot Effects

To resolve temporal changes in the root response, metabolites were classified according to the significance and direction of the R3, R5, and treatment × time contrasts.

Six stringent temporal-response classes were defined:

1. Early-transient: R3 FDR < 0.05, R5 FDR ≥ 0.05, and interaction FDR < 0.05;
2. Late-induced: R3 FDR ≥ 0.05, R5 FDR < 0.05, and interaction FDR < 0.05;
3. Sustained-stable: significant at both R3 and R5 in the same direction, with interaction FDR ≥ 0.05;
4. Sustained-shifted: significant at both time points in the same direction, with interaction FDR < 0.05;
5. Reversed: significant at both R3 and R5 in opposite directions, with interaction FDR < 0.05;
6. Interaction-only: R3 and R5 FDR ≥ 0.05, but interaction FDR < 0.05.

Although root and shoot samples were acquired within the same LC–MS analytical batch, the 5 d tissue comparison was based on within-tissue treatment effect sizes rather than raw peak areas. This approach was chosen because the objective was to compare the responsiveness of the two tissues to the conditioned filtrate, rather than their baseline metabolite abundances. Analyses were restricted to the 4452 metabolites with valid statistical estimates in both tissues.

R5 and P5 log_2_FC values were compared using both Pearson and Spearman correlation analyses. Metabolites were further classified as root-significant only, shoot-significant only, shared same direction, or shared opposite direction according to their BH-FDR status and effect direction.

Overlap between root and shoot DAM sets was assessed using Fisher’s exact test. The expected overlap under independence and the Jaccard index were also calculated to quantify the extent of shared responses. As a sensitivity analysis, concordance between the 5 d root and shoot responses was additionally evaluated using the limma moderated *t*-statistics, which incorporate both estimated treatment effects and empirical-Bayes-moderated feature-wise uncertainty. This analysis was used only to assess the robustness of the cross-tissue concordance conclusion and did not replace log_2_FC as the primary measure of treatment effect.

### 4.8. KEGG Over-Representation and Integrative Analyses

KEGG over-representation analysis (ORA) was recalculated using the DAM sets defined by the final limma analysis [31]. For each comparison or response category, the statistical background was restricted to metabolites that were testable in the corresponding analysis and had at least one KEGG pathway annotation, rather than the full set of 4546 putatively annotated metabolites.

Pathway over-representation was evaluated using a one-sided hypergeometric test. For a given pathway, the enrichment probability was calculated as

(4) $$P(X\geq k)=\sum_{i=k}^{\min(K,n)}\frac{\left(\begin{array}{c} K\\ i \end{array}\right)\left(\begin{array}{c} N-K\\ n-i \end{array}\right)}{\left(\begin{array}{c} N\\ n \end{array}\right)}$$

where *N* denotes the total number of testable metabolites with KEGG pathway annotations in the relevant statistical background; *K* is the number of those background metabolites assigned to the pathway of interest; *n* is the number of KEGG-mapped metabolites in the selected response set; and *k* is the number of metabolites in the selected response set that are assigned to the pathway of interest. Thus, *P*(*X* ≥ *k*) represents the probability of observing at least *k* pathway-associated metabolites in the selected set under random sampling from the defined background. Formal ORA testing was restricted to pathways represented by at least two background metabolites (*K* ≥ 2).

All pathways meeting this criterion were included in Benjamini–Hochberg (BH) multiple-testing correction, including pathways with zero selected hits (*k* = 0). Pathways with BH-FDR < 0.05 were considered to have pathway-level statistical support. Pathways with nominal *p* < 0.05 but BH-FDR ≥ 0.05 were described only as exploratory over-representation tendencies. Response categories containing fewer than five KEGG-mapped selected metabolites were retained in the complete analysis but were not emphasized in the main ORA panels.

To summarize the overall direction of pathway-associated DAMs, a directional abundance score (DA score) was calculated as

(5) $$\mathrm{DA}=\frac{N_{\mathrm{up}}-N_{\mathrm{down}}}{N_{\mathrm{up}}+N_{\mathrm{down}}}$$

where *N*_up_ and *N*_down_ denote the numbers of significantly increased and decreased DAMs assigned to the pathway, respectively. When *N*_up_ + *N*_down_ > 0, the DA score ranges from −1 to 1. Positive values indicate that pathway-associated DAMs were predominantly increased, whereas negative values indicate that they were predominantly decreased. Larger absolute values indicate a stronger directional imbalance. The DA score was used only to describe the directional composition of pathway-associated DAMs and was not interpreted as evidence of pathway activation or inhibition.

Finally, stage-specific differential-abundance results, temporal-response classifications, 5 d tissue effect comparisons, and KEGG annotations were integrated to identify a nonredundant set of representative metabolites for evidence synthesis and visualization. Representative metabolites were selected only after completion of all inferential statistical analyses and therefore did not contribute to significance testing, model fitting, or the definition of statistical thresholds.

## 5. Conclusions

This study shows that exposure to an LB46–*C. equisetifolia* interaction-conditioned filtrate was associated with pronounced temporal turnover in root metabolism and strong differentiation between root and shoot responses at 5 days after inoculation. Recurrent patterns involving central carbon, aromatic, and flavonoid-related metabolism identify metabolic contexts for future targeted investigation.

Because the filtrate represents a composite conditioned environment and its bulk physicochemical properties were not characterized, the present data do not identify a specific chemical signal or distinguish metabolite-mediated effects from other medium changes. Likewise, the study does not establish metabolite origin, transport, pathway activation, or a root-gating mechanism. Rather, it defines a time- and tissue-resolved metabolic response architecture for subsequent mechanistic validation. The present study did not assess growth or physiological performance and therefore does not demonstrate biostimulant efficacy.

## Figures and Tables

**Table 1:** UPLC chromatographic gradient used for LC–QTOF-MS analysis.

Time (min)	Flow Rate (mL min^−1^)	A (%)	B (%)
0.0	0.4	95	5
0.5	0.4	95	5
5.5	0.4	50	50
9.0	0.4	5	95
10.5	0.4	5	95
12.0	0.4	95	5

## Data Availability

The raw data supporting the conclusions of this article will be made available by the authors on request. Further inquiries can be directed to the corresponding author.

## Supplementary Materials

The following supporting information can be downloaded at https://www.mdpi.com/article/10.3390/plants15213469/s1. Figure S1: Abundance distributions and sample-level total signal characteristics of the root and shoot metabolomes; Figure S2: Sample correlations and principal component variance structure of the root and shoot metabolomes; Figure S3: KEGG pathway-annotation coverage across metabolite response sets; Figure S4: Analytical reproducibility of pooled quality-control samples.
