# Supplementary material for: Spatiotemporal Metabolic Reprogramming of Casuarina equisetifolia in Response to an Interaction-Conditioned Filtrate of the Plant Growth-Promoting Bacterium LB46
Source: Plants (Basel). 2026 Nov 6;15(21):3469. doi: 10.3390/plants15213469 (PMC13765805; doi:10.3390/plants15213469)
Supplement: Supplementary file 1 [file plants-15-03469-s001.zip › plants-4636015 Supplementary File.pdf]

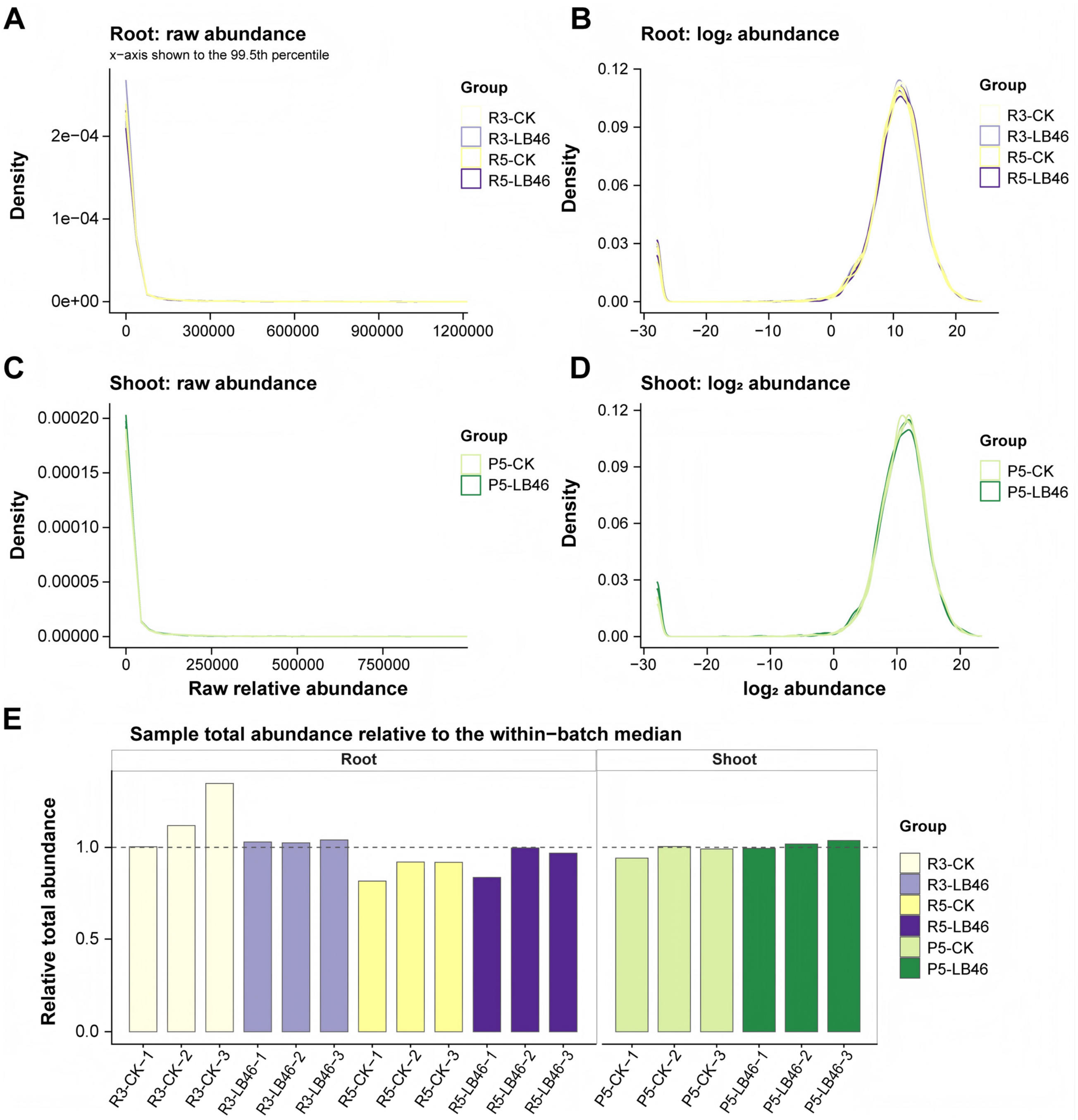

Figure S1. Abundance distributions and sample-level total signal characteristics of the roots and shoots metabolomes. (**A**) Density distribution of raw relative abundances in root samples, with the x-axis truncated at the 99.5th percentile to improve visualization of the main distribution; (**B**) abundance distribution of root samples after $\log_2$ transformation; (**C**) density distribution of raw relative abundances in shoot samples; (**D**) abundance distribution of shoot samples after $\log_2$ transformation; and (**E**) total abundance of each sample relative to the median of the corresponding tissue dataset. R3-CK and R3-LB46 denote the 3 d root control and LB46-conditioned-filtrate treatment groups, respectively; R5-CK and R5-LB46 denote the corresponding 5 d root groups; and the 5 d shoot control and treatment groups, respectively.

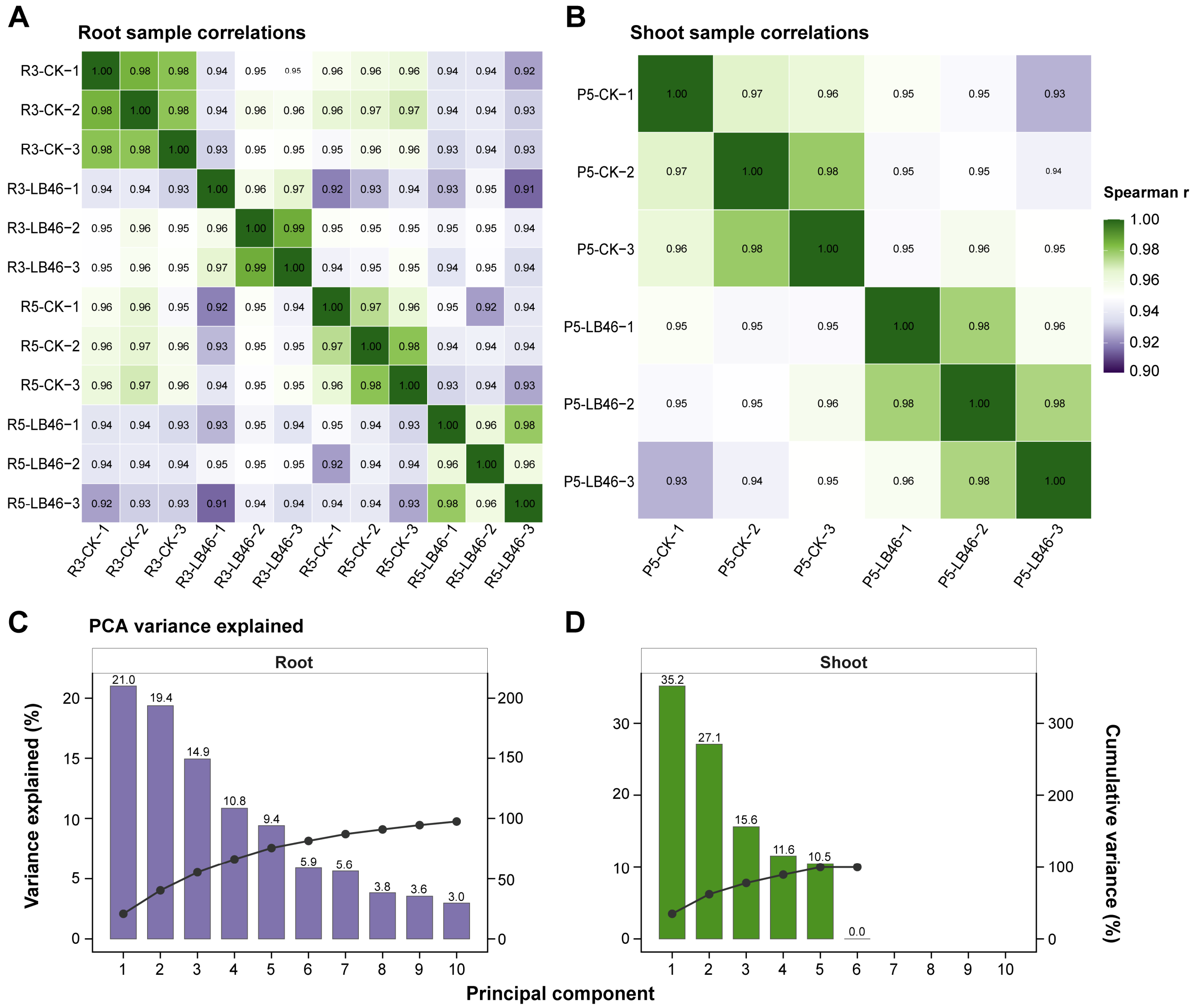

Figure S2. Sample correlations and principal-component variance structure of the root and shoot metabolomes. (**A**) Pairwise Spearman correlation matrix for root samples; (**B**) pairwise Spearman correlation matrix for shoot samples. The color scale ranges from 0.90 to 1.00, and values within cells indicate the corresponding Spearman correlation coefficients. (**C**) Variance explained by the first 10 principal components and cumulative variance explained in the root metabolome; (**D**) corresponding principal-component variance structure of the shoot metabolome. Bars indicate the variance explained by individual principal components, whereas the line indicates cumulative variance explained.

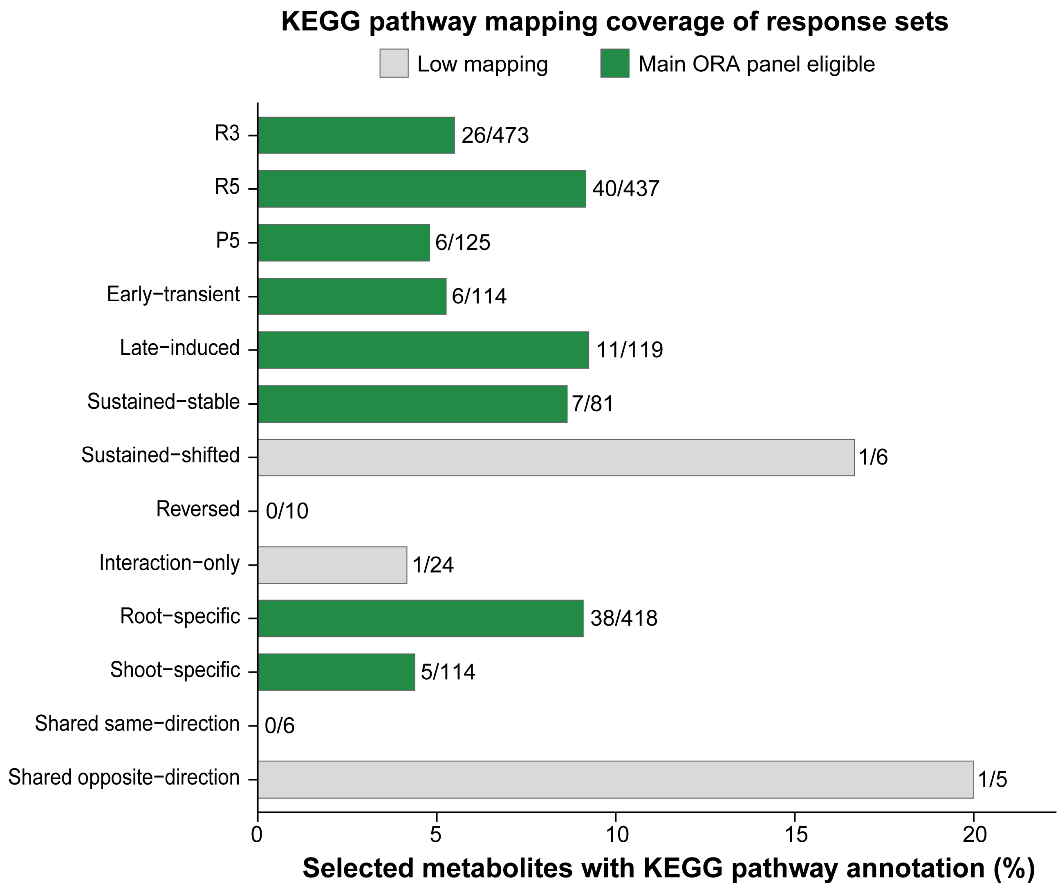

Figure S3. KEGG pathway-annotation coverage across metabolite response sets. Bars indicate the percentage of metabolites with KEGG pathway annotations across stage-specific response sets, strict root temporal-response classes, and 5 d tissue-response classes. Values shown at the end of each bar indicate the number of KEGG pathway-mapped metabolites relative to the total number of metabolites in the corresponding response set. Green bars denote response sets meeting the minimum KEGG-mapping requirement for inclusion in the main ORA panels, whereas gray bars denote sets with low mapping coverage that were retained in the complete analysis but not emphasized in the main ORA figures.

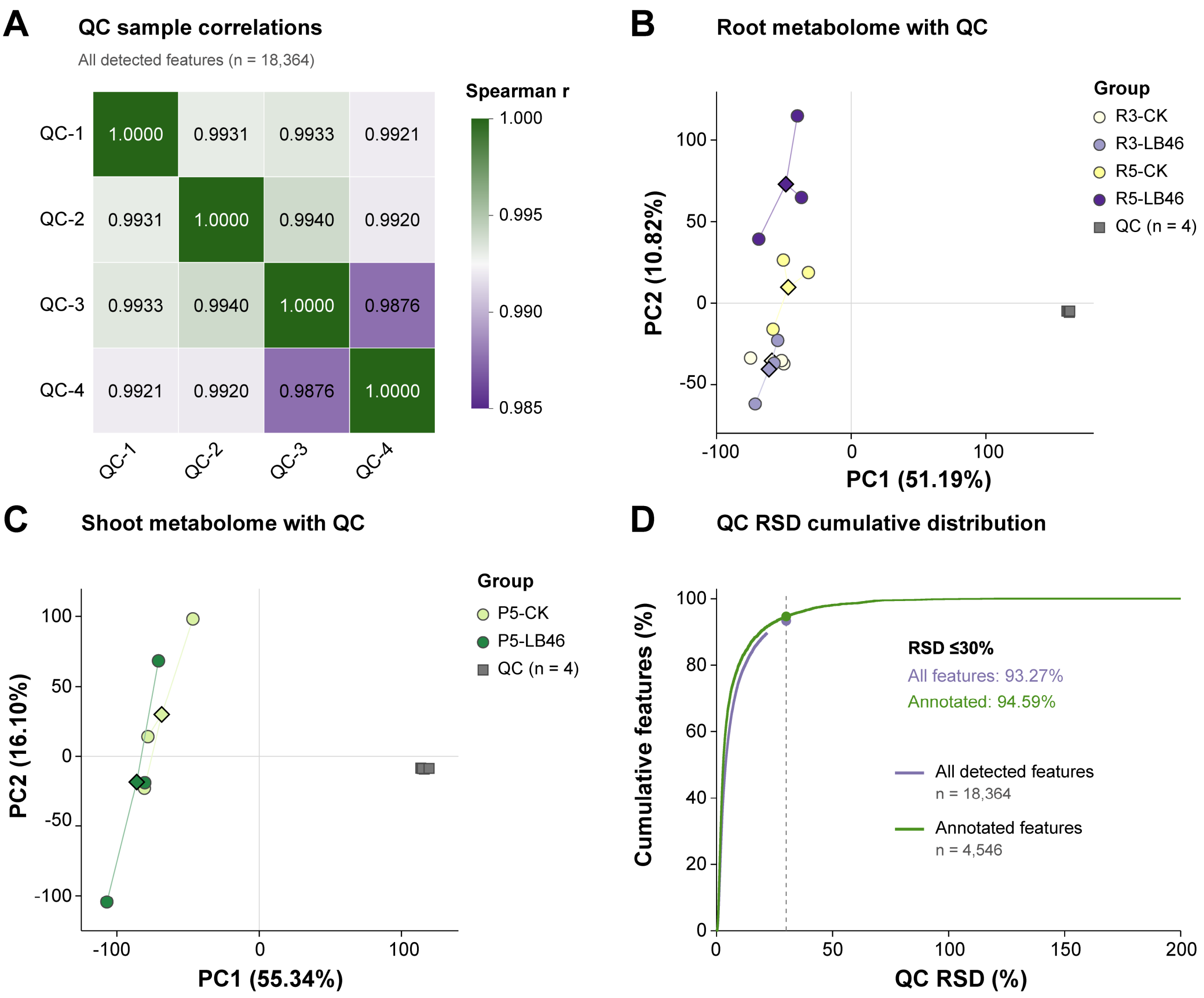

Figure S4. Analytical reproducibility of pooled quality-control samples. (**A**) Pairwise correlation analysis among four pooled QC injections; (**B**) QC-inclusive PCA of the root analytical dataset; (**C**) QC-inclusive PCA of the shoot analytical dataset; (**D**) Cumulative distribution of QC relative standard deviations (RSDs) for all detected features and the 4,546 annotated features used in the present study; dashed lines indicate RSD = 30%.
